# Prevalence and risk factors of bovine viral diarrhea in Colombian cattle

**DOI:** 10.14202/vetworld.2020.1487-1494

**Published:** 2020-08-06

**Authors:** Diego Ortiz Ortega, Rodrigo Alfredo Martínez Sarmiento, Julio César Tobón Torreglosa, Juan Felipe Rocha

**Affiliations:** 1 Corporación Colombiana de Investigación Agropecuaria - AGROSAVIA, Centro de Investigación Tibaitatá, Mosquera, Cundinamarca, Colombia; 2 Empresa Colombiana de Productos Veterinarios – VECOL, Bogotá, Colombia

**Keywords:** bovine, bovine viral diarrhea virus, protective factors, risk factors, seroprevalence

## Abstract

**Background and Aim:**

Bovine viral diarrhea virus (BVDV) is present in most cattle-raising countries around the world, and it has a negative economic impact in cattle herds. In Colombia, previous studies have estimated the prevalence of BVDV in specific locations. The aim of this study was to estimate the prevalence of BVDV in cattle herds located at several municipalities across the country and to identify the associated risk and protective factors.

**Materials and Methods:**

A cross-sectional study was carried out to investigate the prevalence of BVDV in Colombian cattle populations at farm and animal-levels. A total of 387 herds and 8110 animals located in seven different departments were included in this study.

**Results:**

An animal- and farm-level prevalence of 36% and 69%, respectively, were estimated. A high variation for the farm-level prevalence was found among the municipalities studied. Moreover, seropositive cattle to the infectious bovine rhinotracheitis virus (odds ratio (OR)=2.38, p=0.0479) and *Neospora caninum* (OR=3.15, p=0.0122) were more likely to be seropositive for BVDV, while the practice of burning dead animals at the farm was identified as a protective factor (OR=0.17, p=0.014).

**Conclusion:**

The prevalence of BVDV varied more at farm-level compared to animal-level. Two risk factors and one protective factor were identified. The results of the current study are essential to understand the epidemiology of BVDV in Colombia, and to formulate strategies in the region to mitigate the impact of this virus on the productive and reproductive indicators of cattle farms at the regional level.

## Introduction

Bovine viral diarrhea virus (BVDV) is an important pathogen causative of endemic infections in many cattle populations and with a considerable global economic impact. Direct estimated monetary losses due to BVDV in 15 countries during the past 30 years ranged from 0.50 to 687 USD/animal, with higher losses per animal observed in dairy cows (24.85 USD) compared with their beef counterparts [1]. BVDV is a *Pestivirus* with high morbidity and mortality rates associated with an increased premature culling and a decreased reproductive performance [2,3], which is caused by early embryonic death, premature birth, congenital defects, weak calves, stillbirths, and the birth of persistently infected (PI) offspring. The negative effect of BVDV on herd fertility is expressed in longer calving intervals and lower first service conception rates [3]. All these characteristics and events have a significant influence on the monetary level of direct losses in cattle with this infectious condition [1,4]. In Colombia, no official programs exist to control BVDV, and therefore, application of practices to prevent and control the presence of this pathogen varies much and relies on the technical knowledge and the judgment of the producer. Only a few farmers implement immunization as a mitigation method, using multivalent killed vaccines that are commercially available [5].

BVDV is present in most cattle-producing countries worldwide, with at least 88 countries have confirmed infections and 107 countries reporting mitigation activities between 1960 and 2017 [6]. Seroprevalences estimated in regions under the surveillance of the United Nations ranged from 46.23% to 48.73% at the animal level, and from 66.08% to 67.01% at the herd level, with an overall decrease projected for Europe and an increase predicted for North America. For the case of South America, a total of 27 studies allowed the estimation of an average seroprevalence of 53.43% (95% confidence interval [CI=44.19-62.57%]) [2]. In Colombia, most epidemiological studies on BVDV have been carried out at very specific locations such as municipalities or districts within some departments [7-10]. Some of these studies have addressed the identification of risk factors with the available information collected during the sampling phase [11-13]. However, none of these included the diagnose of possible concomitant infection with other pathogens or management and farming practices such as the type of milking, disposal of dead animals, and use of certain veterinarian products that can have an influence on the presence of other immune-suppressive pathogens in the herds.

The aim of this study was to estimate the seroprevalence of BVDV and to identify the associated risk and protective factors in cattle herds located in different departments of Colombia.

## Materials and Methods

### Ethical approval

This study did not need ethical approval. However, it was conducted in accordance with international ethical standards for care and use of animals to minimize stress during the collection of samples.

### Study period, study area, sample size, and study design

The present cross-sectional study was carried out between January 2016 and January 2018. During these 2 years, the epidemiology of the BVD was investigated in 12 municipalities of seven different departments of Colombia. These regions were selected due to their high cattle population density and a high number of cattle farms based on the 2016 livestock census [14]. The sample size was determined using an algorithm to estimate the prevalence of infectious diseases in large populations [15,16]. The number of samples per region was calculated considering the number of animals per municipality and based on a 50% expected prevalence of BVD, a 95% confidence level, and a 3% estimation error. This resulted in a final sample fraction of 6.6%, sampling a total of 8110 non-vaccinated animals distributed in 387 herds.

The samples used for this study were collected as part of a global study to investigate the health status of cattle herds in Colombia. This study included diagnosing other viral, bacterial, and parasitic diseases performed in the same animals that were sampled for BVD, as described below. Results from these tests were included in the analysis to identify possible factors associated with the presence of BVD, as it is described in the data analysis section.

### Sample collection and serological assay

Animals were restrained and handled gently to minimize distress and movement during the collection of samples. In each cow, technicians disinfected the venipuncture site before and after the sampling. Blood samples were collected from the coccygeal vein of cattle using fresh disposable needles and vacutainer tubes with no anticoagulant and were immediately transported to the laboratory. Serum was obtained by centrifuging the tubes with the collected blood to separate the clot. The supernatant was recovered with micropipettes, transferred into 0.5 mL labeled sterile cryovials and stored at −20°C to be analyzed. Detection of antibodies to BVDV was made using the commercial enzyme-linked immunosorbent assay (ELISA) kit INGENESA^®^, which is officially approved by the World Organization of Animal Health (OIE) and it specifically targets the p80/p125 non-structural protein of the BVD virus. This test had a sensitivity of 97.9% and a specificity of 99.7%.

The presence of antibodies against the infectious bovine rhinotracheitis virus (IBRV), enzootic bovine leukosis virus, *Neospora caninum*, parainfluenza type 3 virus (PI3), and bovine respiratory syncytial virus was established using ELISA. Furthermore, *Babesia* parasites were observed by blood smear analysis using Giemsa stain, while *Leptospira* species were detected through microagglutination-lysis reaction.

### Epidemiological BVD data survey

Three hundred eighty-seven selected herds were visited, where a questionnaire and an interview were performed to the producer or the person responsible for the management of the animals. The questionnaire was designed to collect epidemiological data of BVD and other diseases from the cattle farms that comprised this study. Potential risk and protective factors were also included in the questionnaire. These factors had been included in the previous studies of BVD, and some were identified by veterinary epidemiologists. The questions were related to the location of the farm, type of milking, biosecurity practices, feed management, pathologies observed in the herd, characterization of abortions, and others that are described in Table-1.

**Table-1:** Bovine viral diarrhea epidemiological data collected from the questionnaires and interviews conducted among cattle farmers from 12 municipalities in Colombia.

1	Farm identification	
2	Farmer contact details	
3	Farm size and location	
4	Farm (land) tenure	Own/Rented
5	Farm energy supply	Yes/No
6	Predominant breed	
7	Type of milking	Hand/Mechanical
8	Corral for animals	Yes/No
9	Animals from other farms	Yes/No
10	Vaccination plan	
11	Use of individual disposable needle per animal	Yes/No
12	Type of mating	Natural/Artificial insemination/Embryo transfer
13	Bull: Cows ratio	
14	Share bulls with other farms	Yes/No
15	Clinical symptoms in the herd	
	Retained placenta	Yes/No
	Dystocia	Yes/No
	Weak calves	Yes/No
	Joint injuries	Yes/No
	Vulvovaginitis	Yes/No
	Diarrhea	Yes/No
	Fever	Yes/No
	Secretions of mucous membranes	Yes/No
	Mastitis	Yes/No
	Conjunctivitis	Yes/No
	Respiratory disorders	Yes/No
	Progressive weight loss	Yes/No
16	Abortion	Yes/No
	Season	Jan–Mar/Apr–Jun/Jul–Sep/Oct–Dec
	Gestation stage	1^st^/2^nd^/3^rd^ trimester
	Disposal of the placenta and aborted fetus	Burial/Burning/Other
17	Presence of other species	Yes/No
	Dogs	
	Sheep	
	Pigs	
	Horses	
	Birds	
	Buffaloes	
	Wild animals	
18	Disposal of dead animals	Burial/Burning/Other
19	Rodent control	Yes/No
20	Feed storage	Stowage/Bucket/Floor
21	Feed supplements	Hay/Silage/Concentrate/None
	Veterinary supplies	Yes/No
	Technical assistance from a professional	Yes/No
	Mineral and salt supply	
	Average milk yield per animal	
22	Deworming	Yes/No
	Deworming frequency	
	Deworming product applied	
	Soil/Pasture fertilization	Yes/No

### Statistical analysis

The seroprevalence was defined as the presence of antibodies against BVDV in the serum of the host. True prevalence was estimated employing the following equation:


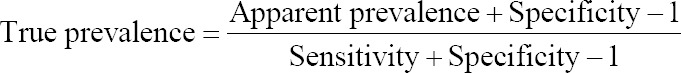


An animal was considered positive when values were equal to or higher than two standard deviations compared with the negative control. A herd was considered positive when antibodies against the virus were found in at least one animal of each farm. Positive (PPV) and negative (NPV) predictive values were calculated to establish the probability of the presence or absence of the disease if the results of the diagnostic tests were positive or negative, respectively. Formulas used to calculate each value are as described by Martin *et al*. [17]:


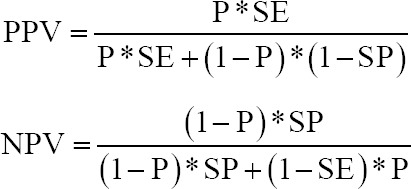


Where P is prevalence, SE is sensitivity, and SP is specificity.

The independent variables were extracted from the survey and data were debugged. Frequency analyses were performed to obtain absolute and relative frequency values for both animals and farms. The ratio of animals and herds affected by BVDV that was exposed to a factor was compared with the same proportion of a non-exposed population to that factor to estimate prevalence ratios (PR). This PR was used to measure the association between BVDV and the hypothetical causal factors, as well as the significance of these associations using a Chi-square test [16]. PR values higher than 1 (lower confidence interval LCI 95% > 1) and with p<0.05 were considered as risk factors, while PR values lower than 1 (upper confidence interval UCI 95% < 1) and with p<0.05 were considered protective factors.

A stratified logistic regression was performed using the Epi-Info 7 software to test for confounding and to identify the simultaneous interaction between the variables significantly associated with BVDV [17]. The homogeneity of variances across treatments was verified using the Bartlett test. When variances showed a homogeneous distribution, statistical significance was determined with the Student’s and Fisher tests. In contrast, when variances were not homogeneously distributed, the non-parametric Kruskal–Wallis and Mann–Whitney-Wilcoxon tests were implemented.

## Results

### BVDV prevalence and incidence rates

A total of 387 farms distributed in 12 municipalities of seven Colombian departments were included and surveyed in this study. Considering that farms with at least one positive animal were classified as positive for BVDV, the overall farm-level prevalence of BVDV was 69.0%. The highest BVDV prevalence in farms was found in the municipalities of Puerto Salgar (100%), Subachoque (92.3%), and Villavicencio (98.6%). On the other hand, the lowest farm-level BVDV prevalence was observed in the municipalities of La Gloria (0%) and Guachucal (23.4%). The mean PPV in farms was 91.5%, while the mean NPV was 83.4% (Table-2).

**Table-2:** Farm-level prevalence and predictive values for BVDV in Colombia.

Department	Municipality	Farms (n)	BVDV positive farms (n)	Apparent prevalence (%)	True prevalence (%)	PPV (%)	NPV (%)
Antioquia	San Pedro de los milagros	29	23	79.3	81.0	99.9	92.5
Boyacá	Sotaquirá	65	53	81.5	83.2	99.9	91.5
Cesar	Aguachica	19	15	78.9	80.6	99.9	92.7
	La Gloria	1	0	0.0	0.0	0.0	100.0
	Rio de oro	8	4	50.0	50.9	99.7	97.9
Córdoba	Monteria	8	7	87.5	89.3	100.0	87.2
Cundinamarca	El rosal	6	4	66.7	68.0	99.8	96.0
	Madrid	5	3	60.0	61.2	99.8	96.9
	Puerto salgar	14	14	100.0	100.0	100.0	0.0
	Subachoque	52	47	90.4	92.3	100.0	83.5
Nariño	Guachucal	151	35	23.2	23.4	99.0	99.4
Meta	Villavicencio	29	28	96.6	98.6	100.0	62.9
Total (n)/ Mean (%)	12	387	233	60.1	69.0	91.5	83.4

PPV=Positive predictive value, NPV=Negative predictive value, BVDV=Bovine viral diarrhea virus.

The analysis of animal-level seroprevalence included a total of 8110 animals (Table-3). In this case, the true BVDV prevalence was 36.6% (3104 positive animals). Antioquia was the department with the highest animal-level BVDV prevalence (73.3%), whereas Nariño showed the lowest BVDV prevalence (24.7%). The mean PPV in animals was 91.1%, while the mean NPV was 98.5%.

**Table-3:** Animal-level prevalence and predictive values for BVDV in Colombia.

Department	Municipality	Animals (n)	BVDV positive animals (n)	Apparent prevalence (%)	True prevalence (%)	PPV (%)	NPV (%)
Antioquia	San Pedro de los milagros	1001	719	71.9	73.3	99.9	94.9
Boyacá	Sotaquirá	1000	374	37.4	38.0	99.5	98.8
Cesar	Aguachica	720	203	28.2	28.6	99.2	99.2
	La gloria	66	0	0.0	0.0	0.0	100.0
	Rio de oro	291	76	25.9	26.5	99.1	99.3
Córdoba	Monteria	1000	398	39.8	40.5	99.5	98.6
Cundinamarca	El rosal	221	72	32.8	33.1	99.4	99.0
	Madrid	80	47	59.0	59.9	99.8	97.1
	Puerto Salgar	1005	319	31.7	32.2	99.3	99.0
	Subachoque	699	391	56.0	57.0	99.8	97.4
Nariño	Guachucal	1027	251	24.4	24.7	99.1	99.3
Meta	Villavicencio	1000	253	25.3	25.6	99.1	99.3
Total	12	8110	3104	36.0	36.6	91.1	98.5

PPV=Positive predictive value; NPV=Negative predictive value, BVDV=Bovine viral diarrhea virus

### Risk and protective factors to BVDV

Data collected from the survey were used to identify risk and protective factors that increased or decreased the likelihood for animals to become infected with BVDV. From a total of 70 factors initially evaluated with Chi-square tests, 11 were established as risk factors and six as protective factors for BVDV. Among the risk factors, fever had the highest association with the presence of BVDV (p=0.000070), whereas mastitis showed the lowest association (p=0.042734). The six protective effect factors were hand milking, concentrate feed supplementation in the diet, burning carcasses of dead animals in the farm, and the use of parasite control treatments such as ivermectin, organophosphates, and cypermethrin (Table-4).

**Table-4:** Risk and protective factors against BVDV in cattle located in 12 municipalities of Colombia determined by Chi-square tests.

Risk factors	Prevalence ratio	95% LCI	95% UCI	p-value
Fever	5.00	1.84	13.61	0.000070
Abortion	4.77	1.75	12.96	0.000120
Bovine leukemia virus	3.24	1.89	5.54	0.000014
Corral	3.02	1.59	5.74	0.000199
Infectious bovine rhinotracheitis virus	2.81	1.63	4.86	0.000131
Mechanical milking	2.78	1.03	7.50	0.017413
*Neospora caninum*	2.52	1.48	4.32	0.001703
Leptospira	2.24	1.32	3.82	0.002263
Burying dead animals	2.23	1.14	4.33	0.008687
Protective factors				
Hand milking	0.41	0.18	0.94	0.0147
Ivermectin	0.37	0.18	0.77	0.0026
Burning dead animals	0.35	0.15	0.82	0.0487
Concentrate feed supplement	0.32	0.18	0.57	0.00002
Organophosphates	0.32	0.17	0.60	0.00011
Cypermethrin	0.25	0.09	0.69	0.0009

LCI=Lower confidence interval; UCI=Upper confidence interval, BVDV=Bovine viral diarrhea virus

After the initial identification of risk and protective factors by performing individual Chi-square tests, a stratified logistic regression was performed to look for significant interactions between these factors and their association with the presence of BVDV. The results showed that the interaction between the presence of IBR virus and the presence of *N. caninum* was significantly associated with the presence of BVDV (p=0.0479 and p=0.0122, respectively) (Table-5). Moreover, the practice of burning carcasses of dead animals on the farm was the only factor with a significant protective effect against BVDV (OR=0.26659; 95% CI=0.0769-0.9194). A second logistic regression, including only the IBR virus and *N. caninum* confirmed the high association of these factors with the presence of BVDV.

**Table-5:** Odds ratios and 95% confidence intervals of risk and protective factors against BVDV in cattle located in 12 municipalities of Colombia.

Risk factors	Logistic regression			

	Odds ratio	Lower limit 95%	Upper limit 95%	p-value
Abortion	6.67	0.73	60.83	0.0923
Corral	0.78	0.19	3.2	0.7278
Burying dead animals	1.39	0.5	3.89	0.5283
Fever	2.84	0.87	9.32	0.0851
Infectious bovine rhinotracheitis virus	2.38	1.01	5.62	0.0479
Ivermectin	0.78	0.15	3.99	0.7627
Leptospirosis	1.37	0.64	2.95	0.4232
Bovine leukemia virus	2.04	0.91	4.56	0.0814
Mastitis	1.36	0.62	3,00	0.4398
*Neospora caninum*	3.15	1.28	7.72	0.0122
Protective factors				
Hand milking	0.74	0.25	21.71	0.585
Ivermectin	0.7	0.23	20.81	0.516
Burning dead animals	0.17	0.04	0.7	0.014
Concentrate feed supplement	0.64	0.24	17.48	0.388
Organophosphates	0.55	0.2	15.47	0.259
Cypermetdrin	0.35	0.1	11.63	0.087

BVDV=Bovine viral diarrhea virus

## Discussion

The true prevalence of BVDV at the animal-level observed in this study (36%) was higher compared to studies carried out in cattle populations of Ecuador (27.0%) [18], Ethiopia (32.6%) [19], and Malaysia (33.2%) [20], but lower than the BVDV prevalence observed in cattle of Egypt (40%) [21] and Bangladesh (51.1%) [22]. In Colombia, the first outbreak of BVD was reported in 1987, which was associated with the import of a group of heifers from the Netherlands in 1975 [5,11]. Moreover, several studies have shown the presence of BVDV in different Colombian regions since then, with BVDV prevalence rates of 29.4%, 46%, 58%, and 27.1% in the Departments of Córdoba, Cesar, Caquetá, and Cundinamarca, respectively [7,8,12,13]. The high BVDV prevalence that was found in this study might be explained by the inclusion of mostly dairy herds in the sampling process. Daves *et al*. [20] found that the prevalence in dairy herds (52.6%) was much higher than in beef herds (7.9%), while the meta-analysis of Scharnböck *et al*. [2] using a sample size of 142,577 individuals also found a higher BVDV prevalence in dairy cattle (51.8%) compared to beef cattle (45.1%). This can primarily be explained by the higher intensive farming present in dairy herds, which involves higher contact frequency between animals and overall higher exposure to the virus [23]. Moreover, most of the animals analyzed in this study were pure Holstein, and some studies have shown that the cattle breed might play an important role. A higher BVDV prevalence was found in pure Holstein Friesian (46.6%) compared to other dairy breeds such as Jersey, Brown Swiss, and a Creole breed (21.8-27.9%) [18]. Likewise, there was a significant effect of the breed on the BVDV seroprevalence of Ethiopian dairy herds, with Holstein Friesian cattle having a higher likelihood (OR=1.3, 95% CI 0.9-1.9%) to be seropositive compared to Jersey cattle [19]. This could indicate the existence of a possible genetic effect on the susceptibility of cows to BVDV. Nonetheless, further studies should include detailed information not only on the type of production system of the sampled animals but also on the breed composition of these, to better evaluate the effect of the animal genetics on the individual and herd level seroprevalences.

Overall, there was a high variation of the farm-level BVDV prevalence rates observed among the different municipalities included in this study (0-100%). It is possible to hypothesize that this considerable variation might be attributed to factors such as geographic barriers, cattle markets, and trade activity, as well as the agroecological characteristics from the region itself, which could have facilitated or prevented the transmission of the virus among farms. However, a similar herd-prevalence was found in the studies carried out by Fernandes *et al*. [24] (65.5%; 95% CI=61.1-69.7%) and Velasova *et al*. [25] (66%; 95% CI=56-77%) in Brazil and Great Britain, respectively. Nevertheless, the variation in the BVDV prevalence in these studies is higher in the latter since the sampling included only one region (state of Paraíba, Brazil), while the former included a stratified random sampling covering almost 95% of all dairy farms in Great Britain. In our study, the sampling covered a wide range of herds across different regions of Colombia, and there was a high variation in the number of herds included per municipality. For instance, in the Department of Cesar, only one negative BVDV herd was sampled in the municipality of La Gloria, meanwhile, in Nariño, 151 farms were included from only one municipality.

The results showed that cattle that were seropositive to IBRV and *N. caninum* were more likely to be seropositive to BVDV. Some studies have shown associations among these pathogens when one or more of these are present in the production system. In British dairy farms, a correlation of 0.21 between the presence of BVDV antibodies and *N. caninum* was found [25]. Likewise, the seroprevalence of BVDV was associated with the seroprevalence to neosporosis in Irish beef herds [26], while the exposure to BVDV was linked to the seroconversion to neosporosis in Canadian dairy cows [27]. Furthermore, positive associations have been found between BVDV, IBRV, and bovine parainfluenza virus Type 3 in indigenous calves in Western Kenya [28], indicating that a seropositive animal to one of these pathogens would have a higher likelihood of being seropositive to any of the other two. In Colombia, the previous studies showed mixed viral infections for BVDV and IBRV [9,10], which may be explained by their high prevalence, some similarities in their routes of transmission, and possibly by sharing some risk and protective factors. Furthermore, some of these microorganisms may have an immunosuppressive effect, which would increase the susceptibility of animals to get infected with pathogens such as BVDV.

Several factors have been associated with the prevalence of BVDV in cattle herds in different countries and regions around the world. Some of these belong to the animal itself, while some correspond to specific characteristics from the herd or the production system. Risk factors from the animal include age, breed, lactation, and pregnancy status [19,20]. Farm-level factors include farming intensity, herd size, type of mating, housing patterns, and even the distance between the manure pit and the farm [18,19,29]. In the present study, some of these were initially identified as risk factors with independent Chi-square tests (e.g., the existence of corrals in the farm, and use of mechanical milking), although none of them were significant under the multivariate logistic regression analysis. The large heterogeneity of epidemiological and mitigation factors among BVDV studies might be attributed to different modeling approaches and to the omission of covariates such as herd immunity, management practices, age of animals, stocking rate, and community pasturing activities [30], which are of remarkable importance in pasture-based systems, the predominant cattle farming strategy in the South American region.

The only significant protective factor identified was the practice of burning dead animals at the farm. The World Organization for Animal Health (OIE) recommends different practices for the disposal of dead animals that include incineration, especially in a dedicated facility where the corpses can be entirely burned and reduced to ash so that inactivation of pathogenic agents occurs [31]. Most cattle farms in Colombia do not have this kind of facility, and the burning of dead animals takes place in open spaces. Despite this, results found that in the current study would suggest that cattle farmers in the country are completing this incineration process in a way that BVDV is successfully eliminated. Moreover, it would also indicate the importance of removing the products of abortions such as fetuses/embryos, placentas, and fluids, a practice that, when applied jointly with the incineration of dead cattle, might prevent the likelihood of transmission and reduce the prevalence of BVDV in the herd.

The studies of Aragaw *et al*. [19] and Kumar *et al*. [29] suggested that small size of the herd might protect against BVDV transmission due to a higher self-clearance. Extensive pastoral farming has also been associated with a lower seroprevalence of BVDV [18], which is explained by the reduced stocking rate on the paddocks and often a lower density of cattle in the facilities of the production system. We did not evaluate these variables in our study; however, we expected to find other protective factors such as the use of individual disposable needles per animal or a closed herd status. This might be due to the difference in times of implementation and the level of strictness with which these biosecurity measures were applied in the farms included in the study. In the meta-analysis made by Pinior *et al*. [30], the authors discuss several factors that could affect the effectiveness of mitigation measures for BVDV and its significance as protective factors, including failures during vaccination, selection of inappropriate culling strategies, and difficulty to distinguish between infection and re-infections.

It is important to highlight the absence of official programs to control BVDV in the region. Besides monitoring and surveillance, prevention and mitigation activities are essential for the control of BVDV, which includes implementation of vaccination programs at regional and national levels [32,33]. Several studies have measured the efficacy of BVDV vaccination to prevent BVD transmission and reduce its impact on productive and reproductive parameters. The efficacy of available KV varies significantly, something that is attributed to the strains chosen, inactivation techniques, antigen mass, and/or adjuvants used [4]. In Colombia, BVDV vaccines are inactivated and are sold in combination with other viral and bacterial antigens, including IBRV. Nevertheless, a negligible proportion of farmers use it because of its high cost and due to the fact that these diseases are not included in nationwide disease eradication programs. Further studies on the immune response to different commercially available vaccines in the region are required, as well as monitoring antibody titers at different times after vaccination to determine and compare its efficacy.

Most studies on BVDV in Colombia and throughout the region used antibody tests to estimate the prevalence and to identify risk factors. However, immunological tests cannot differentiate between vaccinated and naturally infected animals; therefore, interpretation of antibody tests is sometimes tricky in regional epidemiological studies [2]. Besides immunological tests, future studies should measure additional laboratory parameters such as levels of circulating white blood cells and platelets, detection of antigens, and clinical parameters that indicate the level of protection and response acquired through vaccination, such as pyrexia, pregnancy, abortion, and live offspring born rates.

## Conclusion

The BVDV prevalence found is within the range of other studies carried out in Colombia, but higher than in other regions. There was a higher variation of the prevalence found at the farm-level than at the animal-level. The presence of the bovine rhinotracheitis virus and *N. caninum* in the herd increased the likelihood for an animal having BVD, while the practice of burning dead animals at the farm was a protective factor against BVDV. Further studies are required to continue the surveillance of this pathogen in the region and to compare the efficacy of different BVDV control methods in the country.

## Authors’ Contributions

DOO conceived the research, organized the fieldwork, analyzed and interpreted the results, and drafted the manuscript. RAMS contributed to statistical analysis and data interpretation. JCTT designed the concept for this research, administered the questionnaires, and made data interpretation. JFR participated in the analysis of data, interpretation, and discussion of the results. All authors read and approved the final manuscript.

## Competing Interests

The authors declare that they have no competing interests.

## Publisher’s Note

Veterinary World remains neutral with regard to jurisdictional claims in published institutional affiliation.
